# Vitamin D Therapy May Induce Lipoma Involution: A Multi-case Report

**DOI:** 10.7759/cureus.74412

**Published:** 2024-11-25

**Authors:** Lydia Martin, Raphaella Lambert, Susan Hoadley, Layla Shadman, Raphael C Lee

**Affiliations:** 1 Plastic Surgery, The University of Chicago Medicine, Chicago, USA; 2 Plastic Surgery, Pritzker School of Medicine, Chicago, USA

**Keywords:** adipose, benign lipoma, regression, surgical excision, vitamin d

## Abstract

Benign lipomas are a common medical problem that is not known to regress spontaneously. In addition, vitamin D (VD) is a known regulator of adipocyte proliferation and differentiation. Thus, the purpose of this multi-case study was to determine if optimizing serum 1,25(OH)D3 (VD3) concentrations to the 40-60 ng/mL range would catalyze regression of benign subcutaneous lipomas. This IRB-approved study was interrupted by the COVID-19 epidemic. Nine patients presenting to the plastic surgery clinic before the pandemic and who followed up after defined the study cohort. Patients underwent lipoma size measurement and serum 25(OH)D (VD) assay at the clinic visit before and after the pandemic. Enrolled patients were prescribed 10,000 IU of VD3 intake daily for three months if serum VD levels were abnormally low and then 5,000 IU daily thereafter. Patients were prescribed 5,000 IU daily if serum VD levels were within a normal (30-40 ng/dL) range. The treatment duration was seven to 18 months. Of the nine patients who were compliant with oral VD3 intake and exhibited increased serum VD levels, six manifested substantial lipoma size regression, and three manifested lipoma growth arrest. Serum VD levels increased in all (nine) patients compliant with VD3 treatment. Although limited in size, this study suggests that VD3 therapy possibly promotes lipoma regression and should be further investigated.

## Introduction

Lipomas are the most common benign mesenchymal neoplasm found in adult humans [1,2], and in the United States, lipomas are the most prevalent subcutaneous neoplasm, having an incidence of 2.1 per 1000 population [3]. Most superficial lipomas manifest as subcutaneous, soft, mobile, and well-encapsulated tumors, and their origin can be deep or superficial relative to the fascial layers. They can also present as multiple small tumors with fascial attachments. Histologically, most superficial lipomas consist of slow-growing, mature, and well-differentiated adipocytes. Benign lipoma phenotypes also include hemangiolipomas and spindle cell lipomas. Treatment recommendations range from monitoring growth and managing symptoms to surgical excision. Since lipomas are a very common health concern, they are a significant public health problem.

Common reasons for lipoma excision include functional limitations, aesthetic impairments, discomfort, and concerns about the risk of malignancy [1,3]. For benign lipomas, surgical excision with histopathological verification is the traditional definitive approach to management [1]. Suction aspiration through a small incision is another method used to remove lipomas, and clinical monitoring for increases in growth kinetics is also a common medical management strategy [2]. The morbidity associated with such surgical interventions includes scarring, nerve injury, and soft tissue deformity [2].

Vitamin D (VD) is known to regulate adipogenesis and adipocyte differentiation [4-7]. Many in-vitro studies using 3T3-L1 mouse pre-adipocytes report an inhibitory effect of VD on adipocyte differentiation [4-6]. In all, 1,25(OH)2D3 is known to bind to the nuclear VD receptor (VDR) with high specificity and affinity, followed by heterodimerizing with the retinoid X receptor. Adipogenesis proceeds through a series of steps requiring regulation of select transcription factors, starting with CCAAT/enhancer-binding protein (C/EBP) β and C/EBPδ expression, followed by activation of nuclear receptor peroxisome proliferator-activated receptor γ (PPARγ) and C/EBPα, which leads to an increase in triglyceride formation. When bound to 1,25(OH)2D3, VDR attenuates C/EBPβ mRNA and C/EBPβ nuclear protein levels at an important phase early in differentiation when C/EBPβ is needed to induce C/EBPα and PPARγ [8]. Further, evidence suggests that 1,25(OH)2D3 inhibits C/EBPα and PPARγ, both of which are necessary for maintaining adipocyte differentiation in addition to adipogenesis [9]. Also, sub-physiological concentrations of VD3 are known to inhibit adipocyte apoptosis [5]. Moreover, biological studies reveal a substantial link between VD deficiency and adiposity due to both enhanced parathyroid levels and increased calcium flow into adipocytes, thereby increasing lipogenesis [10]. Human studies indicate that VD has a direct effect on adiposity [11,12].

In our clinical practice, VD3 deficiency, if it exists, is corrected prior to performing elective surgery for multiple reasons, including optimizing both skin immunity and the patient’s stress responses. We observed that two VD3 deficient patients presenting for forehead lipoma excision experienced substantial lipoma regression in response to VD3 therapy and no longer required excision. Thus, with these clinical observations and the established role of VD in regulating adipogenicity, we postulated that VD3 therapy may induce lipoma involution and reduce the need for surgical intervention.

## Materials and methods

**Figure 1:**
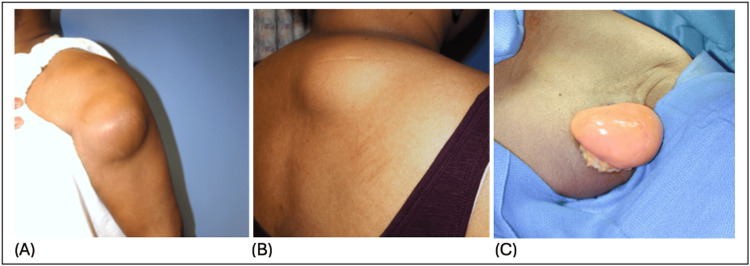
Subcutaneous lipoma presentation (A,B) Photographs illustrating the clinical presentation of the subcutaneous lipomas in our study group. (C) Characteristic intra-operative appearance of a lipoma in our clinic with a similar history and clinical presentation as those in our study population.

The University of Chicago Institutional Review Board (IRB) approval was obtained (IRB approval name “Vitamin D3 & Lipoma” and approval number IRB19-1023) to investigate the effect of oral VD3 supplementation in patients that underwent subcutaneous lipoma removal (Figure 1). Inclusion criteria for enrolled patients included those ≥18 years of age with a clinical diagnosis of solitary superficial subcutaneous lipoma and no evidence of lipoma-related syndromes, specifically for those who were not already taking VD3 supplements. Exclusion criteria included those <18 years of age, those with a serum 25(OH)D level >50 ng/dL, and/or those with a history of hypercalcemia or hyperparathyroidism. Patients presenting for evaluation of their lipoma in the clinic underwent routine pre-operative testing for 25(OH)D (VD) concentration that reflects both VD2 and VD3 serum concentrations. The threshold level for reliable serum VD assay was approximately 7 ng/dL.

**Figure 2:**
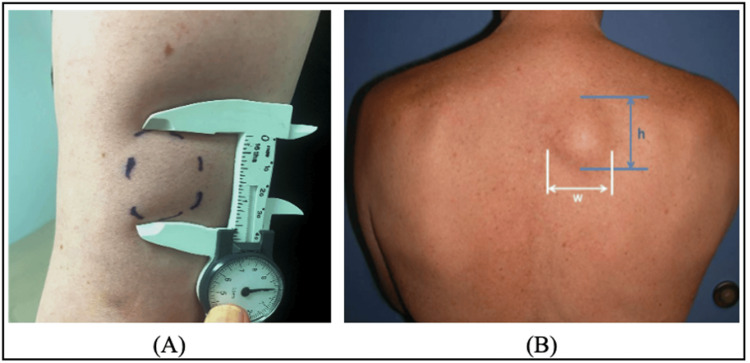
Lipoma measurement technique The technique was used to measure the maximal biplanar dimensions of lipomas in this study. Care was taken not to identify the maximal projection of the lipoma in the subcutaneous space. Simultaneous measurement of opposite extensions of the lesion was made to avoid displacement of the mobile tumor. (A) Representation of lipoma dimension evaluation with calipers. (B) Representation of the anatomical transverse and longitudinal directions used for measurement.

Patients were prescribed daily 10,000 IU VD3 supplementation in tablet form (United States Pharmacopoeia (USP) certified) if serum 25(OH)D levels were less than 30 ng/dL (ng/dL = nanograms/deciliter). If serum concentrations of VD were within 30-50 ng/mL, patients were prescribed 5,000 IU VD3 daily. Lipoma dimensions were measured with calipers at initial evaluation using transverse and craniocaudal plane measurements; the caliper was positioned at the outer maximal edge of the lipoma so that skin marks could then be applied to the largest projection of the lipoma on the skin in both the anatomical transverse and longitudinal directions. This was reproduced for subsequent measurement (Figure 2). We performed or directly supervised all lipoma measurements to avoid deforming the lipoma during the measurements and to maintain consistency in measurement.

According to the protocol, the enrolled patients were to be scheduled for follow-up evaluations at three-month intervals, at which time the lipoma dimensions and serum VD3 levels were to be measured again. The study was interrupted by the COVID-19 epidemic, and the outpatient clinic was closed to non-urgent medical conditions. Once the clinics were reopened for patients with benign medical conditions, 10 patients scheduled follow-up appointments.

The change in bi-directional measurement of the lipomas was recorded as either discernably increased (↑), decreased (↓), or unchanged (↔), with the latter indicating a measured change in lipoma size as less than 2.5 cm² or if one of the length or width measurements increased in size post-VD3 treatment, being therefore possibly unreliable. Follow-up was interrupted by the COVID-19 pandemic, during which time the follow-up was carried out by video conferencing. After the pandemic, patients were given the option to continue VD3 therapy or undergo surgical resection of the mass.

## Results

Due to the COVID-19 pandemic, only nine patients completed the protocol, and enrollment was aborted for nine patients. The majority of patients had measurable changes in lipoma size following VD3 supplementation. We report the findings from these patients as a multi-case report without statistical analysis.

Nine patients indicated that they were compliant with the study’s VD3 supplementation protocol. The patients were predominantly female (six of nine) and African American (seven of nine). The average age at presentation was 59 years (range = 39-76 years). Two-dimensional lipoma size measurements and serum VD levels were recorded from patients who obtained follow-up measurements after the COVID-19 pandemic, and they are listed in Table 1.

**Table 1:** Patient data: demographics, serum VD responses, and lipoma size changes Demographics from hospital medical records. Gender: M, male; F, female Race: B, Black; W, White VD, vitamin D; DM, diabetes mellitus type 2; BMI, body mass index; ↓, decreased; ↔, no measurable change; Rx, therapy; cm, centimeters; ng, nanograms; dL, 100 mL

Patient	Duration of study (months)	Pre-VD_3_ Rx serum (VD) (ng/dL)	Post-VD_3_ Rx serum (VD) (ng/dL)	Pre-VD_3 _Rx lipoma size (cm)	Post-VD_3_ Rx Lipoma size (cm)	Change in lipoma size (cm)	Patient age (years)	Gender	Race	DM	BMI >30
1	18	39	79	5 x 5	3 x 2	↓	67	F	B	+	-
2	13	12	39	3 x 3	2 x 1.6	↓	58	F	B/W	-	-
3	10	26	32	5 x 3	3 x 4	↔	41	M	B	-	-
4	11	~7	26	2.5 x 2.5	1.7 x 2.1	↓	39	F	B	-	-
5	8	15	42	2 x 4	2.1 x 3.4	↔	67	F	B	-	-
6	7	~7	46	2 x 2.5	1.8 x 1.5	↔	76	M	B	-	-
7	11	9	27	5.5 x 4.5	2.2 x 4.1	↓	62	M	B	-	+
8	9	16	28	6 x 6	5.1 x 3.5	↓	60	F	W	-	+
9	12	25	43	13 (diameter)	9 x 7	↓	61	F	B	+	-

As revealed in Table 1, a decrease in lipoma dimensions was measured in six of the nine patients compliant with their VD3 treatment recommendation. The lipoma sizes in three patients were recorded as unchanged based on the criteria stated in the methods section. The average duration of VD3 treatment was 11 months. An increased serum VD level was achieved in all compliant patients treated with VD3 at 10,000 IU daily and all patients with documented VD insufficiency (VD <21 ng/mL) prior to VD3 supplementation. In addition, a tenth patient did not comply with the VD3 treatment recommendation and exhibited an increase in lipoma size; this patient was only interested in lipoma surgical resection. No detectable side effects or complications of VD3 therapy were reported by any participants during the course of the study.

## Discussion

Small subcutaneous lipomas are usually painless and seldom require surgical excision. However, when concern for malignancy, discomfort, pain, or issues of cosmesis arise, surgical excision of the mass is the most commonly employed treatment. In addition, while liposarcomas are very rare, patients with enlarging lipomas are often referred for surgical excision due to uncertainty of the tumor’s diagnosis and malignant potential. It is important to note that in the United States, lipoma treatment has a substantial impact on healthcare expenditures. Ambulatory surgery statistics from the Center for Disease Control’s (CDC) National Center for Health Statistics (NCHS) indicate that approximately 100,000 ambulatory surgical procedures are performed annually in the United States to remove lipomas [13]. However, as for any surgical procedure, excision has the potential for associated surgical complications, including hematoma, surgical site infection, peripheral nerve injury, skeletal muscle injury, and cosmetic deformities, all of which highlight the need for better, less invasive, non-surgical treatments.

Given the well-established effects of VD on adipogenesis and involution, it would be surprising if mature adipocyte proliferation and apoptosis did not depend on adipose tissue VD concentrations. This might include benign lipomas consisting of mature adipocytes. Of course, there are several different phenotypic variations of benign lipomas and some variability of these types in their regulation by VD3.

In addition, the appropriate VD levels in serum have become a high-priority question for medical research. The optimum VD serum concentration is a subject of considerable debate [14]. Some investigators recommended that the level of circulating VD be around 20-30 ng/mL and no more than 50 ng/mL [15]. It could well be that the optimum serum VD level depends on the health status of the patient. Since it is widely appreciated among health care providers that VD is an important immunomodulator that coordinates the activity of various modes of immune defense in humans [4-6], patients with immunological disorders or who are under challenge by physiological stress or infection may benefit from different than normal serum VD levels.

Moreover, it has been reported that population mean VD levels vary with lifestyle and geography [16]. Mean serum 25(OH)D concentrations were significantly lower in urban than rural populations living in northern African countries, in the country of South Africa compared to sub-Saharan African countries, in women compared to men, and in newborn babies compared to their mothers [16]. These data also correlate with the link between indoor living and sunlight exposure. Human populations have existed on Earth for more than 100,000 years, and the transition to indoor working and living has been relatively recent. Overall, healthy optimum serum VD levels over a range of conditions remain to be determined [17].

In this study, six patients had VD3 levels lower than 20 ng/mL. After adding the 10,000 IU VD3 supplement, an increase in VD3 level over 20 ng/mL was achieved in all compliant patients. Consistent with our initial expectations, we also found a decrease in the size of the evaluated lipoma measured in the majority of compliant patients treated with VD3, and there were no detectable adverse side effects or complications related to the treatment. Moreover, corrective VD3 treatment could potentially have significant public health benefits, given both the burden of lipomatous disease and the probability of a widespread VD deficiency [14,18].

Study limitations 

Due to the COVID-19 pandemic, only nine patients were part of the study cohort. In addition, since these findings are part of a multi-case report, there are no controls. Therefore, further investigation is warranted. Last, using calipers as a measurement tool provided a surface level measurement; however, in future studies, magnetic resonance imaging or ultrasound should be considered as they are more detailed and accurate, especially for deeper lesions.

## Conclusions

The results from this multi-case report suggest that administering corrective VD3 treatment to patients with benign, soft, subcutaneous lipomas may arrest lipoma growth and possibly promote involution. Controlled clinical trials are warranted. If validated in a larger clinical study, VD3 supplementation may be a cost-effective and medically appropriate treatment for benign subcutaneous lipomas.
